# Identification of key genes controlling L-ascorbic acid during Jujube (*Ziziphus jujuba* Mill.) fruit development by integrating transcriptome and metabolome analysis

**DOI:** 10.3389/fpls.2022.950103

**Published:** 2022-08-04

**Authors:** Dongye Lu, Yang Wu, Qinghua Pan, Yuping Zhang, Yuanyong Qi, Wenhui Bao

**Affiliations:** Institute of Forestry and Pomology, Beijing Academy of Agriculture and Forestry Sciences, Beijing, China

**Keywords:** *Ziziphus jujuba*, L-ascorbic acid, metabolomics, transcriptomics, transcriptional regulation, *ZjERF17*, *ZjbZIP9*, *ZjGBF4*

## Abstract

Chinese jujube (*Ziziphus jujuba*) is a vital economic tree native to China. Jujube fruit with abundant L-Ascorbic Acid (AsA) is an ideal material for studying the mechanism of AsA biosynthesis and metabolism. However, the key transcription factors regulating AsA anabolism in jujube have not been reported. Here, we used jujube variety “Mazao” as the experimental material, conducted an integrative analysis of transcriptome and metabolome to investigate changes in differential genes and metabolites, and find the key genes regulating AsA during jujube fruit growth. The results showed that AsA was mostly synthesized in the young stage and enlargement stage, *ZjMDHAR* gene takes an important part in the AsA recycling. Three gene networks/modules were highly correlated with AsA, among them, three genes were identified as candidates controlling AsA, including *ZjERF17* (LOC107404975), *ZjbZIP9* (LOC107406320), and *ZjGBF4* (LOC107421670). These results provide new directions and insights for further study on the regulation mechanism of AsA in jujube.

## Introduction

Plant primary metabolites include sugars, amino acids, lipids, nucleotides, vitamins, and other substances, which are essential basic substances and energy sources in life activities such as plant growth, development, and reproduction. Among them, L-Ascorbic acid (AsA, also known as vitamin C) is one of the important metabolites and standards to evaluate the nutritional quality and commercial value of vegetables and fruits. As an important antioxidant, it participates in the defense mechanism of plants, plant adversity, biotic, and abiotic stress response, and other biological processes (Smirnoff, 2011). AsA is also a class of organic substances necessary to maintain human life activities, which can prevent and inhibit the occurrence of cancer, prevent scurvy, lower blood cholesterol, and enhance the immune system (Carr and Frei, 1999; Pawlowska et al., 2019). Humans cannot synthesize AsA by themselves due to the loss of the function of L-gurono-γ-lactone oxidase (Fransson and Mani, 2007). The acquisition of AsA mainly depends on fresh vegetables and fruits.

The AsA content of tissues depends on its biosynthesis and recycling. There are four biosynthetic pathways of AsA in plants: L-galactose (D-mannose) pathway (Wheeler et al., 1998), Myo-inositol pathway (Lorence et al., 2004), D-galacturonate pathway (Agius et al., 2003), and L-gulonate pathway (Wolucka and Van Montagu, 2003). Among them, L-galactose pathway is the most important synthetic pathway. In strawberry, apple, kiwifruit, *Rosa roxburghii* Tratt, tomato, and *Arabidopsis thaliana* (L.) Heynh., genes such as GDP-D-mannose-3, 5-epimerase (*GME*), GDP-L-galactose phosphorylase (*GGP*), and L-galactose dehydrogenase (*GaLDH*), as key genes of this biosynthetic pathway, influence the changes of AsA content (Li et al., 2009, 2010; Massot et al., 2012). In AsA recycling, AsA is produced by a reduction of oxidized forms of AsA (mono- and dehydroascorbate), which maintains the content of AsA (Alós et al., 2013).

Chinese jujube (*Ziziphus jujuba* Mill.), which belongs to the *Ziziphus* genus of Rhamnaceae, is a commercial tree native to China with strong stress resistance, wide suitable range, rich nutrition, and high medicinal value (Liu et al., 2015). Jujube fruit is called a “natural vitamin C pill.” The AsA content in fresh jujube is between 200 and 1,000 mg/100 g FW, which is five times that of strawberry, 20-fold that of citrus, and more than 70 times that of apple. However, the research on AsA in jujube fruit stays in measurement and description (Gan et al., 2002; Zhao et al., 2006; Bai et al., 2016; Zhang et al., 2016), or the dynamic changes of some AsA synthesis pathway genes during fruit development (Zhang et al., 2016). It is reported that there are two AsA biosynthesis pathways (L-galactose and Myo-inositol pathways) in jujube (Liu et al., 2014; Huang et al., 2016), but there are few in-depth studies on the molecular regulatory mechanisms of AsA in jujube, especially, transcription factors, or transcriptional regulation mechanism.

In recent years, multi-omics combined analysis and quantitative biology have become an effective means to explore gene networks and their regulatory mechanisms related to phenotypic traits (Zhang et al., 2019, 2022; Li S. et al., 2021). Among them, weighted gene co-expression network analysis (WGCNA) is a powerful and widely used method for identifying modules/networks of co-expressed genes based on mRNA-Seq data, linking these modules to phenotypic traits, and detecting candidate regulatory genes in the networks (Langfelder and Horvath, 2008). For example, Umer et al. (2020) identified seven genes that are highly associated with sugars and organic acids in watermelon by using WGCNA. This method has also been applied in the quality research of apples, apricot, and other fruits (Bai et al., 2015; Zhang et al., 2019). Furthermore, the joint of transcriptome and metabolome allows accurate co-expression analysis of differentially expressed genes (DEGs) and differentially accumulated metabolites (DAMs) in time series. Combined with biological function analysis such as functional annotation and metabolic pathway enrichment, key metabolic pathways, genes, and metabolites can be targeted to systematically analyze the association between plant regulation mechanisms and biomolecular functions. Many studies were reported about transcriptome and metabolome combined analysis, such as the synthesis pathway of flavonoids, lignans and anthocyanin (Chen C. Y. et al., 2020; Li S. et al., 2021) or molecular regulatory mechanisms under salt or drought stress (Shi et al., 2020; Zhang et al., 2022).

Recently, improving fruit nutritional quality and tolerance have attracted extensive attention. Therefore, it is necessary to systematically understand the molecular regulation mechanism of AsA in jujube. In this research, we integrated jujube metabolomic and transcriptome data to (1) detect the changes of differential genes and metabolites during the development of jujube fruit; (2) explore the potential transcription factors related to AsA biosynthesis, and (3) reveal the associated transcriptional regulatory networks. The findings of this study would deepen our understanding of AsA biosynthesis mechanisms and provide valuable insights for mining potential transcription factors.

## Materials and methods

### Plant materials

**FIGURE 1:**
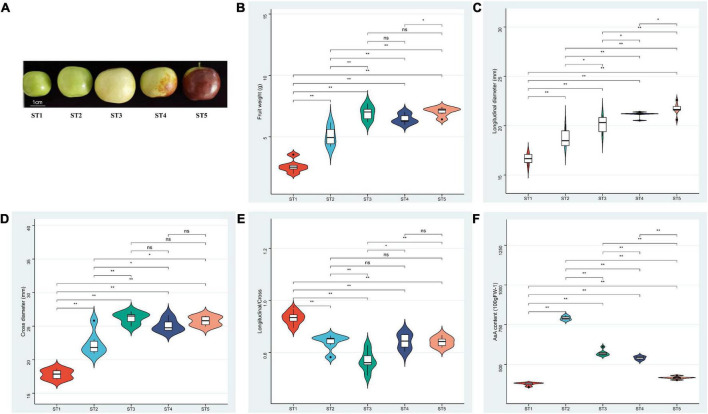
Phenotype traits of “Mazao” from ST1 to ST5 stages. **(A)** Shape. **(B)** Fruit weight. **(C)** Longitudinal diameter. **(D)** Cross diameter **(E)** Longitudinal/Cross. **(F)** AsA content. The statistical analysis was determined using Ducan’s mutiple range test, *P < 0.05; **P < 0.001; ns means Not significant.

One of the Chinese jujube excellent cultivars “Mazao” was used in this study, and it was selected from Hebei province and cultivated in Qinglonghu town (116°5′E, 39°47′N), Fangshan District, Beijing, China. “Mazao” is fresh jujube with an oblate shape and high acidity flavor (Figure 1A), We collected fruit samples from all developmental stages, including young [ST1, 30 days after anthesis (DAA)], enlarged (ST2, 60 DAA), white-ripened (ST3, 80 DAA), half-red (ST4, 100 DAA), and fully red (ST5, 110 DAA) fruits. The pulp was enucleated, cut into small pieces, frozen in liquid nitrogen and stored at –80°C. At each stage, three biological repeats were conducted for metabolomic analysis and transcriptomic sequencing. At the same time, the fruit weight and fruit size (longitudinal and cross diameter) during fruit development were also recorded, and at least 20 fruits were measured for each character.

### Determination of ascorbic acid content

AsA was extracted in metaphosphoric acid. The specific experimental operation is carried out according to Zhang’s method (Zhang et al., 2016). We weighed 0.5 g of fresh jujube fruit, put it into a pre-cooled mortar after peeling, and quickly added 3 ml of 0.2% metaphosphoric acid to grind. The homogenate was centrifuged at ∼13,400 × g for 15 min at 4°C (5804R, Eppendorf, CA, Germany). The supernatant was diluted to 10 mL with 0.2% metaphosphoric acid for AsA determination. Total AsA levels were determined using the method described by Gökmen et al. (2000). The content of jujube AsA was determined by high-performance liquid chromatography (HPLC) system. The mobile phase was composed of 15% methanol and 85% metaphosphoric acid aqueous solution, pH 2.5. The flux was set to 1 ml/min and the injection volume was 10 μl. The column temperature was set at 35°C. The spectra were obtained at wavelength 243 nm. All samples were analyzed in triplicate.

### Metabolomic analysis

The 100 mg vacuum freeze-dried powder of biological samples was weighed and dissolved in 1.2 mL of 70% methanol solution at 4°C overnight. After centrifugation (centrifugal forces ∼13,400 × g, 10 min) and filtration, the sample was used for UPLC–ESI-MS/MS analysis. All primary metabolites were annotated by the self-built database NWDB (Metware Biotechnology Co., Ltd., Wuhan, China) and quantified using multiple reaction monitoring (MRM). Analyst 1.6.1 software (AB Sciex) was used to analyze metabolite data which were log2-transformed and normalized in statistical analysis to improve normality. To identify DAMs, we used |log2(fold change)| ≥ 1 and variable importance in project (VIP) ≥ 1 as the screening criteria. We also used Venn diagrams to illustrate the number of differential metabolites. Principal component analysis (PCA) and KEGG analysis were performed to understand the metabolic pathways in the five fruit stages of jujube.

### Ribonucleic acid sequencing and data analysis

Total ribonucleic acid (RNA) of jujube fruits at five stages was extracted from frozen fruits samples using the RNAprep Pure Plant kit (DP441, Tiangen, China) and DNAse digestion was performed to remove DNA; Illumina RNA-Seq by Metware Technologies Co., Ltd. (Wuhan, China). Sequencing libraries were generated using NEBNext^®^ Ultra™ RNA Library Prep Kit according to the manufacturer’s instructions. The raw data for fifteen RNA-seq were quality- controlled by fastp v0.19.3 software. After reads filtering, clean reads were obtained and mapped to the jujube reference genome *Z. jujuba* Mill. ‘‘*Dongzao’’* (accession number: SAMN02918186)^1^ (Liu et al., 2014) by using HISAT v2.1.0 (Kim et al., 2015). We used StringTie v1.3.4d for new gene predictions. FeatureCounts v1.6.2 was applied to calculate the gene alignment (Liao et al., 2014), then the FPKM (fragments per kb per million reads) values of each fragment were analyzed by using RESM software (Li and Dewey, 2011) based on the gene length. DESeq2 v1.22.1 (Love et al., 2014) was used to identify DEGs between the pairwise comparisons and corrected the *P*-values using Benjamini and Hochberg’s method. Adjusted *P*-values < 0.05 and |log2(fold change)| ≥ 1 were used as thresholds for significant differential expression. We performed Gene Ontology (GO) and the Kyoto Encyclopedia of Genes and Genomes (KEGG) databases analysis for the DEGs (Mao et al., 2005).

### Phenotype-gene correlation analysis

WGCNA was performed with default parameters in R, and the gene was simplified into co-expressed modules (Zhang and Horvath, 2005; Langfelder and Horvath, 2008). The FPKM values were normalized and the adjacency matrix was constructed. The metabolite data produced from the metabolome [including vitamins, organic acids, saccharides and derivatives, glycerol ester, nucleotides, and derivatives, Lysophosphatidyl cholines (LPC), Lysophosphatidyl ethanolamines (LPE), and free fatty acids] were also considered as phenotypic data and were imported into the WGCNA package to calculate correlation-based associations between phenotype and gene modules with default settings. The adjacency matrix is transformed into a topological overlap matrix (TOM) by the WGCNA package. After constructing a network, the genes with a high degree of co-expression were grouped into a module with default parameters, and eigengenes for these modules were calculated. Cytoscape software version 3.8.0 was used to visualize the interaction network of phenotypes and genes. To better reveal the key candidate genes, we performed a correlation analysis of the screened genes with AsA and its related genes using the R package “corrplot.”

### qRT-PCR

Total RNA of the samples was extracted by an easy fast plant tissue RNA extraction kit (Cat. No. DP452, TIANGEN, China). The RNA concentration and quality were detected with a NanoDrop-2000 Analyzer (Thermo Fisher Scientific Inc., Waltham, United States). The synthesis of the first-strand cDNA was conducted by using a cDNA Reverse Transcription Kit (PrimeScript™ RT Master Mix, Takara). The primers used in this study were listed in Supplementary Table 1. All of the reactions of qRT-PCR analysis were carried out by using TB Green^®^ Premix Ex Taq™ II (Cat. No. RR820A, TakaRa) and acted on the CFX96™ Real-Time System following the manufacturer’s instructions. We selected the *UBQ* (NCBI accession number: EU916200.1) gene as the internal reference gene (Zhang et al., 2016), and the 2^–ΔΔ^*^CT^* relative quantification method (Livak and Schmittgen, 2001) was used to analyze the relative expression level. We conducted three independent biological replicates and three technical replicates.

## Results

### Phenotypic analysis and ascorbic acid metabolic profiling during fruit development

Our previous investigation indicated that the material “Mazao” (Figure 1A) selected in this study has high AsA content, soluble solid content of 22.1%, and folic acid content of 35.5 μg/100 g. It is a variety with a sweet and sour taste, good quality and high edible rate. As shown in Figures 1B,D, in the process of fruit development, the changing trend of weight and cross diameter was consistent, and both showed an increase in ST1 and ST2 stages and tended to be stable at the ST3 (white-ripening stage) of the fruit. An increase in weight was observed from ST2 to ST3. But the longitudinal diameter and aspect ratio of the fruit showed a trend of increasing all the time and first increasing and then decreasing, respectively (Figures 1C,E).

To study the content and accumulation trend of AsA during the development of jujube fruits, we measured the content of AsA in five development stages of jujube using HPLC method. There were significant changes in AsA measurements at different developmental stages. The concentration of AsA was extremely high at ST2 with 795.95 mg/100 g FW but decreased at maturity (Figure 1F).

### Metabolomic analysis

**FIGURE 2:**
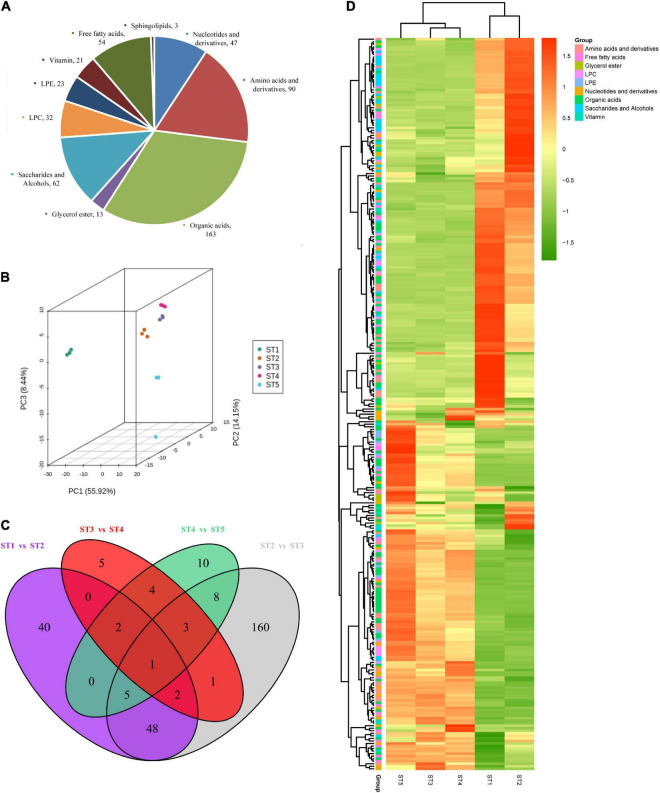
The metabolomics data of the jujube fruit at different stages. **(A)** Pie chart of primary metabolites identified in jujube fruits. **(B)** PCA analysis of the jujube fruit samples. **(C)** Venn diagram showing the differential accumulated metabolites (DAMs) of comparison groups. **(D)** Heatmap of 228 differentially accumulated metabolites (DAMs). Different colors in the heat map represent the values obtained by normalizing the relative content of differential metabolites, reflecting their relative content (the color bar from green to red represents low content to high content), and the annotation bar on the right side of the clustering tree corresponds to the secondary substance of Class II.

To investigate the changes in metabolites of jujube at different stages, primary metabolite analysis of jujube fruits at five stages was performed to uncover the metabolites based on LC-MS/MS. A total of 508 metabolites were detected and classified into 10 categories in this study, including 163 organic acids which were the most enriched types, 47 nucleotides and derivatives, 13 glycerol esters, 90 amino acids and derivatives, 62 saccharides and alcohols, 32 LPCs, 23 LPEs, 21 vitamins, 54 free fatty acids, and three sphingolipids (Figure 2A). The results of PCA analysis indicated that the samples from each group were completely separated, and the axis 1, 2, and 3 principal components explained 78.51% of the total variation (Figure 2B).

To further explain the metabolites differences in the developmental stage, a total of 289 metabolites were DAMs during pairwise comparison (Supplementary Table 2), the most divergent were ST2 vs. ST3, which contained the highest number (228) of differential metabolites (Figure 2C). Among them, 123 DAMs were down-regulated while 105 DAMs were up-regulated (Supplementary Table 3). We found that only one DAM (L-Tryptophan) was shared by ST1 vs. ST2, ST2 vs. ST3, ST3 vs. ST4, and ST4 vs. ST5 (Figure 2C). As shown in Figure 2D, the cluster analysis of identified DAMs from five stages exhibited a clear grouping pattern: Most DAMs from ST1 and ST2 clustered with a highly significant expression which implied ST1 and ST2 were the key time of metabolites formation in jujube fruit development. The AsA we are concerned about is also among them (Supplementary Figure 1).

### Transcriptomic analysis during jujube fruit development

RNA-seq was performed to understand the transcriptome changes of jujube fruit at different stages. The transcriptome data of these 15 samples have been deposited in NCBI Sequence Read Archive (SRA) database with accession number: PRJNA835207. A total of 711,747,180 raw reads were obtained, after filtering, 685,429,928 clean reads were matched to the genome with a mapping rate of 87.24–88.81%, and the GC percentages ranged from 43.53 to 44.21% (Supplementary Table 4). We used the FPKMs to express the number of transcripts identified in each sample, as shown in Supplementary Table 5, the FPKM values of ST1 and ST2 samples were higher than other stage samples.

**FIGURE 3:**
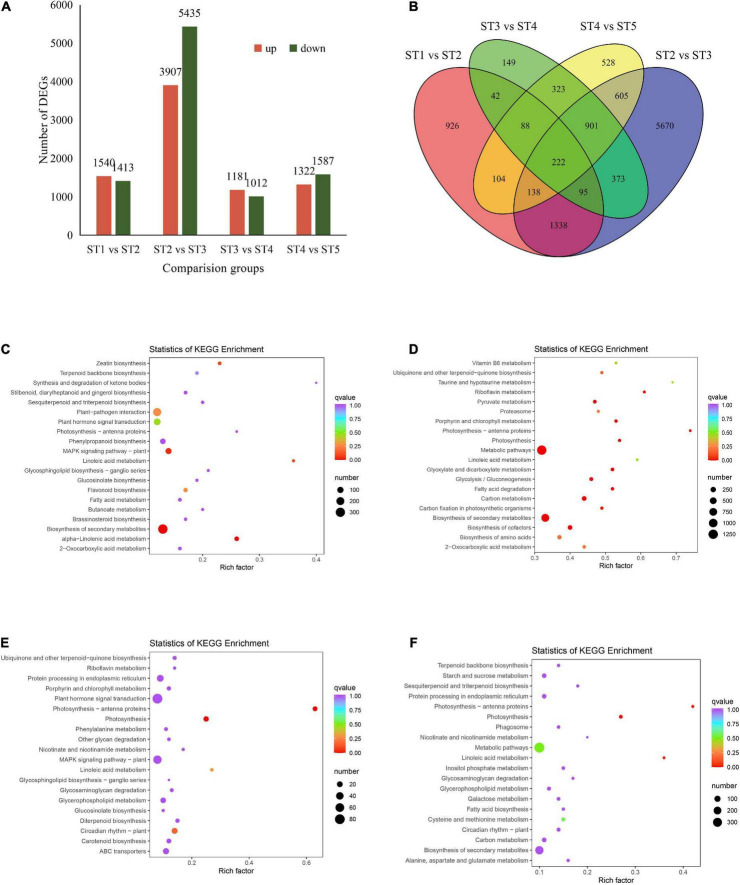
DEGs involved in jujube fruit at different developmental stages. **(A)** Number of DEGs identified in comparison groups. **(B)** Venn diagram of DEGs in four comparison groups. **(C–F)** Represents KEGG enrichments in each pairwise comparison, respectively. The vertical coordinate represented the corresponding rich factor for each pathway, the horizontal ordinate represents the name of the pathway, the color of the dots reflects the *p*-value, and the redder the more significant the enrichment. The size of the dots represents the number of enriched differential genes.

The transcripts from five developmental stages of jujube fruit were grouped into four comparisons, the DEGs, GO, and KEGG pathway enrichment was analyzed. The results showed that there were 2,953, 9,342, 2,193, and 2,909 DEGs in ST1 vs. ST2, ST2 vs. ST3, ST3 vs. ST4, and ST4 vs. ST5, respectively (Figure 3A), among them, ST2 vs. ST3 group contained the most up-regulated (3,907) and down-regulated (5,435) DEGs (Supplementary Figure 2 and Supplementary Table 6). There were 222 genes shared by the four comparative combinations (Figure 3B). For GO analyses, the top 50 DEGs were mostly enriched in the category of biological process in jujube fruit development (Supplementary Figure 3). It is worth noting that in the ST2 vs. ST3 pairwise comparison, the biological processes, cellular components, and molecular function types involved in the differential genes were significantly higher than in other comparisons (Supplementary Figure 3). Furthermore, KEGG enrichment analysis revealed that the DEGs identified in the first 20 pathways act on secondary metabolite biosynthesis, metabolic pathway, MAPK signaling pathway, photosynthesis, and plant circadian rhythm (Figures 3C–F).

### Expression of genes related to ascorbic acid biosynthesis and recycling

**FIGURE 4:**
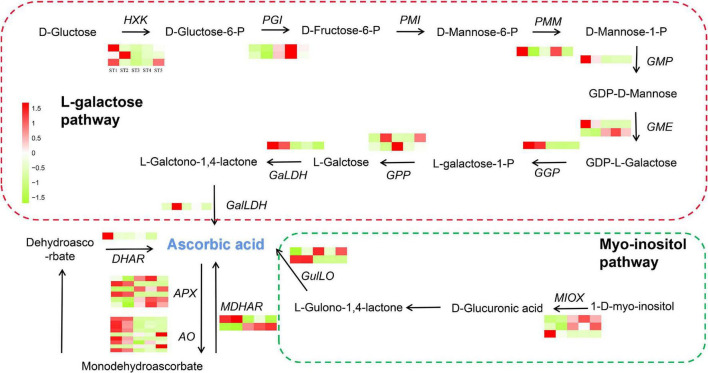
The map of AsA biosynthetic and recycling pathway.

Firstly, we identified the genes of AsA synthesis and recycling pathways, referring to the reports from jujube “Dongzao” (Liu et al., 2014). Secondly, based on the transcriptome data, we drew a heat map of the DEGs associated with AsA synthesis and recycling during jujube fruit development (Figure 4). Finally, in this study, we identified 15 genes associated with AsA synthesis (11) and AsA recycling (4), which are *hexokinase* (*HXK*), *D-glucose-6-phosphate isomerase* (*PGI*), *phospho-mannomutase* (*PMM*), *GDP- mannose pyrophosphorylase* (*GMP*), *GDP- mannose-3′,5′-epimerase* (*GME*), *GDP-L-galactose* (*GGP*), *L-galactose-1-phosphate phosphatase* (*GPP*), *L-galactose dehydrogenase* (*GaLDH*) in the galactose synthesis pathway, respectively. *L-galactono-1,4-lactone dehydrogenase* (*GalLDH*), *L-gulono-1,4-lactone oxidase* (*GulLO*), and *myo-inositol oxygenase* (*MIOX*) genes in the inositol pathway, *ascorbate peroxidase* (*APX*), *ascorbate oxidase* (*AO*), *dehydroascorbate reductase* (*DHAR*), and *monodehydroascorbate reductase* (*MDHAR*) genes in the AsA recycling (Figure 4). The results of heat map analysis indicated that most of these genes reached their highest expression levels during the ST1 and ST2 stages of jujube fruit development.

### Weighted gene co-expression network analysis correlation network analysis of gene modules related to ascorbic acid accumulation

**FIGURE 5:**
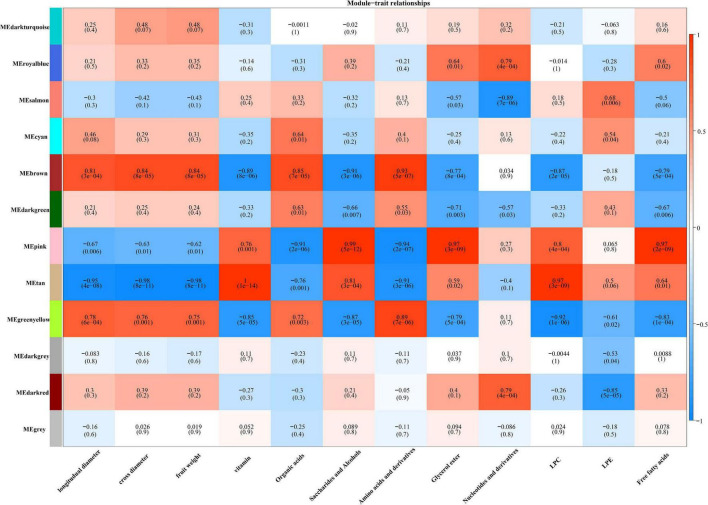
Module-trait correlations. The color scale represents *r*^2^-values from –1 (blue) to 1 (red).

To find key transcription factors associated with the accumulation of AsA, we performed a WGCNA analysis using the WGCNA R package based on phenotypic trait data. There were 12 modules with similar gene expression patterns (Figure 5). The results indicated that the genes from the MEtan module were highly positively correlated with vitamins with a correlation coefficient of 1, while genes included in the MEbrown and MEgreenyellow modules were negatively correlated with vitamins, with correlation coefficients of 0.89 and 0.85, respectively (Figure 5). Among them, we selected candidate genes from each of these three modules based on the annotation information of the reference genome of jujube “Dongzao.”

**FIGURE 6:**
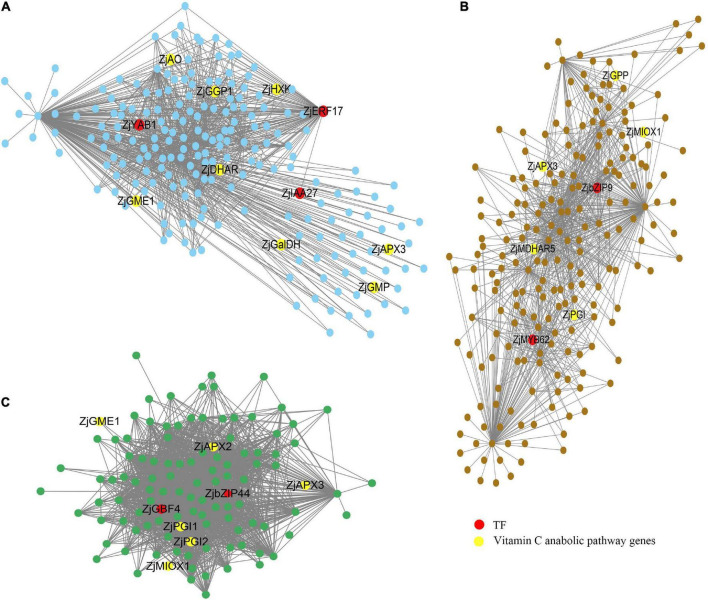
Gene networks involved in AsA regulation during jujube fruit development. **(A)** Gene network for the MEtan module. **(B)** Gene network for the MEbrown module. **(C)** Gene network for the MEgreenyellow module.

Cytoscape software was used to visualize the edges and nodes of candidate genes which were calculated from the WGCNA R package. As shown in Figure 6, we selected three hub genes [*Ethylene-responsive transcription factor ERF17* (*ZjERF17*) (LOC107404975), *Axial regulator YABBY1* (*ZjYAB1*) (LOC107403723), and *Indoleacetic acid-induced protein 27* (*ZjIAA27*) (LOC107404288)] from MEtan modules, two hub genes [*Basic leucine zipper 9* (*ZjbZIP9*) (LOC107406320) and *Myb-related protein 62* (*ZjMYB62*) (LOC107403759)] from MEbrown module, and two hub genes [*Basic leucine zipper 44* (*ZjbZIP44*) (LOC107424569) and *G-box-binding factor 4* (*ZjGBF4*) (LOC107421670)] from MEgreenyellow module as the candidate transcription factors with red color. Among them, *ZjERF17*, *ZjbZIP9*, and *ZjGBF4* are the most probable hub genes with high connectivity values.

L-galactose pathway genes (*ZjHXK*, *ZjPGI*, *ZjGMP*, *ZjGME*, *ZjGGP*, *ZjGPP*, *ZjGalDH*) and degradation related genes (*ZjAO*, *ZjAPX*) were found in all three modules, *ZjMIOX* genes in inositol pathway were found in MEbrown and MEgreenyellow module, and MEtan and MEbrown module contain recycling pathway genes *ZjDHAR* and *ZjMDHAR*.

In addition, we added the correlation analysis of these three candidates with AsA content and AsA synthesis-related genes. The results are shown in Supplementary Figure 4. The correlation coefficients of three candidates *ZjERF17*, *ZjbZIP9*, and *ZjGBF4*, with AsA were 0.99, –0.92, –0.89, respectively. They also showed high correlation with some AsA synthesis-related genes. The above results suggest that *ZjERF17* (LOC107404975), *ZjbZIP9* (LOC107406320), and *ZjGBF4* (LOC107421670) are most likely to be important genes regulating AsA synthesis.

### qRT-PCR analysis of differentially expressed genes

**FIGURE 7:**
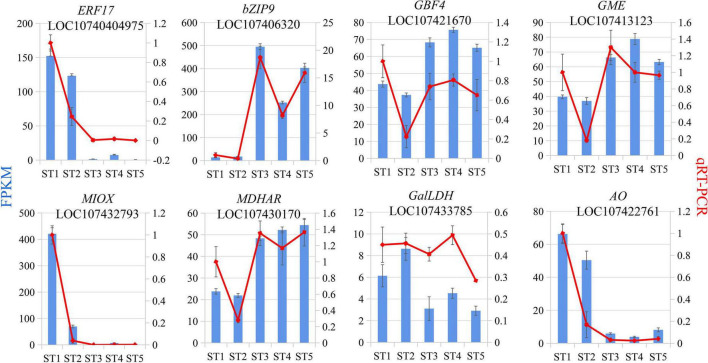
The qRT-PCR validation results.

To test the credibility of our transcriptome data and to lay the foundation for functional verification of transcription factors to be mined later, including transcription factors identified from co-expression networks, eight genes involved in the AsA biosynthesis at five fruit stages were selected for qRT–PCR verification. The primers’ information of these genes was shown in Supplementary Table 1. The qPCR results exhibited that the expression patterns of these 8 genes were consistent with those of transcriptome results (Figure 7).

## Discussion

### Genetic mechanism of ascorbic acid changes during jujube fruit development

AsA can function as a cofactor for certain reactions, as an antioxidant- eliminating free radicals, or as a donor or receptor for electron transport chains in plasma membrane and chloroplasts, thus affecting many aspects of plant growth and development (Fenech et al., 2019). AsA accumulation in plants consists of synthesis, degradation, and recycling and is a complex compound biological process (Fenech et al., 2019). Previous studies have shown that the synthesis pathway and accumulation trend of AsA in plant species are various. For example, the L-galactose pathway and D-galacturonic acid pathway were found in strawberries, and these two pathways played an alternate role in maintaining AsA in immature and mature strawberries, respectively (Cruz-Rus et al., 2011). Both grape and tomato possess three pathways except for the L-gulonate pathway (Melino et al., 2009; Badejo et al., 2012), and during the development, the AsA content of tomato fruit increased continuously from the veraison stage to full maturity (Ioannidi et al., 2009). There are two AsA biosynthesis pathways: L-galactose and inositol pathways in jujube (Liu et al., 2014), but the content of AsA in jujube was much higher than in the above species, suggesting that the levels of AsA are related to the degradation and recycling capacity of each plant rather than the number of synthetic pathways. Besides, our results showed that AsA content accumulated obviously in the young fruit stage (ST1) and enlargement stage (ST2) of jujube “Mazao” fruit development, and decreased after white ripening stage (ST3) (Figure 1F), which was consistent with previous study of jujube “Junzao” (Zhang et al., 2016). A similar accumulation pattern was observed in kiwifruit (Li et al., 2010). In contrast, the AsA content of peaches decreased with fruit development (Imai et al., 2009).

For DEGs in AsA synthesis, as shown in Figures 4–6, the MEtan module contains the most genes in the jujube AsA synthesis pathway and recycling synthesis pathway, including *ZjGGP*, *ZjGME*, *ZjGMP*, *ZjGalDH*, *ZjHXK*, *ZjDHAR*, *ZjAO*, and *ZjAPX*. Among them, *ZjDHAR*, *ZjAO*, and *ZjAPX* were genes involved in the recycling process, while the other five genes were acted on AsA synthesis, and most of these genes were highly expressed in ST1 and ST2 stages (Figure 4 and Supplementary Table 5), which was consistent with the changes in AsA content (Supplementary Table 2 and Supplementary Figure 1). The above results suggest that these genes have an important effect on maintaining high AsA content during the early development of jujube fruit. There are two AsA synthetic pathways in jujube, and Huang’s study based on the proteomic level of jujube identified 15 proteins involved in the L-galactose pathway but no proteins related to the myo-inositol pathway (Huang et al., 2021). However, notably, in our study, since the expression levels of the two isoforms of *ZjMIOX* genes and *ZjGulLO* gene in myo-inositol pathway was up-regulated from the ST3 (white-ripening stage) (Figure 4), indicating that the inositol pathway may play a vital role in maintaining AsA accumulation at maturity. These results were in agreement with the findings of Zhang’s research on jujube (Zhang et al., 2016). Furthermore, we found that *ZjMDHAR* is a crucial gene in the recycling pathway, and its two isomers play a complementary role in transforming AsA during the whole development period of fruit, which indicates to a great extent that *ZjMDHAR* is also the key gene to maintaining high AsA in jujube. All these results proved that jujube fruit accumulated high AsA through strong synthesis and recycling synthesis ability, ST1 and ST2 stages are the key processes involved in AsA biosynthesis and cycling of jujube.

The semi-red and full red stages are periods of interest to consumers, so increasing the AsA content of jujubes can help increase their economic value and nutrition. However, according to our results, the actual AsA content was decreased during these two periods, during which the expression of *MIOX* genes related to the myo-inositol pathway was increasing at a later stage. According to the literature, many cis-acting elements related to light response elements, defense and stress response elements were identified in the promoter of *MIOX* (Li Z. et al., 2021). In addition, it has also been shown that ultraviolet-C (UVC) light irradiation of postharvest acerola fruit significantly reduces AsA degradation (Rabelo et al., 2020). We therefore infer that light regulation may be a beneficial measure to maintain AsA content in jujubes for ripening.

### Candidate transcription factors identified in ascorbic acid pathways

Gene expression regulation is one of the hotspots in modern molecular biology, and the regulation of transcription level is the key to gene expression regulation. However, the transcriptional regulation of AsA has been mainly studied in model plants, such as tomatoes and *A. thaliana* (Mellidou et al., 2021). Previous studies have reported transcription factors like *SlNFYA10* (Chen W. et al., 2020), *SlHZ24* (Hu et al., 2016), *SlZF3* (Li et al., 2018), *SlbHLH59* (Ye et al., 2019), *ABI4* (Kakan et al., 2021), *AtERF98* (Zhang et al., 2012), and *MdERF98* (Ma et al., 2022). They not only regulated AsA biosynthesis but also improve plant resistance by binding to the promoter regions of structural genes such as *GMP1*, *GMP2*, *GMP3*, and *PMM* in the L-galactose pathway (Zhang et al., 2012; Hu et al., 2016; Li et al., 2018; Ye et al., 2019; Chen W. et al., 2020; Ma et al., 2022). As an important medicinal and edible fruit tree with high AsA content, the transcriptional regulation mechanism of AsA in jujube is still unclear.

In the present study, we screened and predicted seven candidate genes by WGCNA analysis (Figure 6), and the homologous genes of these genes have been reported to be related to plant resistance in other species. For example, Overexpression of *ERF17* in cauliflower (*Brassica oleracea* L. var. *botrytis* L.) significantly improved the tolerance of transformants to drought and heat stress (Li H. et al., 2021); *AtbZIP9* affected seed germination under saline conditions by regulating mitochondrial *AtTrxo1* (Ortiz-Espín et al., 2017); *VvMYB62* can improve the germination rate of transgenic *A. thaliana* and enhance the salt tolerance of plants (Wang, 2014); *GBF4* was identified in drought response of rice by meta-analysis of quantitative traits loci (QTL) (Selamat and Nadarajah, 2021); The abiotic stress-related gene *bZIP44*, was activated by GmANK114 under drought and salt stresses in soybean (Zhao et al., 2020).

*VcIAA27* was highly expressed in the early stage of blueberry fruit development, suggesting that *VcIAA27* may take an important part in fruit expansion (Hou et al., 2020). Similarly, the rapid accumulation of AsA in jujube fruit during the fruit expansion period, and there may be some internal relationship between them. Furthermore, *StYABBY1* can reverse regulate chlorophyll accumulation and photosynthesis (Wu, 2019), and the accumulation of AsA in plant cells or tissues and organs is also regulated by light (Mastropasqua et al., 2012). In conclusion, all seven genes identified in the study were candidate regulating factors while *ZjERF17* (LOC107404975), *ZjbZIP9* (LOC107406320), and *ZjGBF4* (LOC107421670) were the hub genes with high connectivity values. The results of this paper will provide a valuable reference for the in-depth study of AsA mechanism in jujube.

## Conclusion

In the present study, transcriptome and metabolite analyses of jujube fruits were performed to detect genes and metabolites change during jujube fruit development and to explore gene networks (based on co-expression patterns) regulating AsA pathways. We found that genes associated with AsA anabolism are highly expressed in early fruit development. *ZjMDHAR* played a vital part in AsA recycling. The high content of AsA in jujube depends on the high expression of related genes in the synthesis and recycling pathway. We identified three AsA-related gene networks/module co-expression patterns and identified seven genes involved in AsA-controlled- metabolism, three of which were hub genes. These genes are newly discovered, and their role in jujube has not been reported before.

## Data availability statement

The data presented in the study are deposited in the SRA repository, accession number PRJNA835207.

## Author contributions

QP and YZ conceived the research. DL conducted experiments, analyzed data, and wrote the manuscript. YW revised the manuscript. YQ and WB collected samples. All authors have read and agreed to the published version of the manuscript.

## Footnotes

1
https://www.ncbi.nlm.nih.gov/genome/?term=JUJUBE

## Supplementary material

Supplementary Figure 1 Cluster heatmap of 13 differentially accumulated vitamin-related metabolites.

Supplementary Figure 2 Volcano plots of DEGs between different jujube fruit development. The X-axis and the Y-axis represent the fold change of DEGs and the significance level of DEGs, respectively.

Supplementary Figure 3 GO analysis of DEGs in ST1 vs. ST2 **(A)**, ST2 vs. ST3 **(B)**, ST3 vs. ST4 **(C)** and ST4 vs. ST5 **(D)**. The X-axis and the Y-axis indicate GO classifications and the number of genes in each classification, respectively.

Supplementary Figure 4 Correlation analysis between AsA content and related genes.
