# Supplementary figures and images for: Identification of key genes controlling L-ascorbic acid during Jujube (Ziziphus jujuba Mill.) fruit development by integrating transcriptome and metabolome analysis
Source: Front Plant Sci. 2022 Aug 4;13:950103. doi: 10.3389/fpls.2022.950103 (PMC9386341; doi:10.3389/fpls.2022.950103)

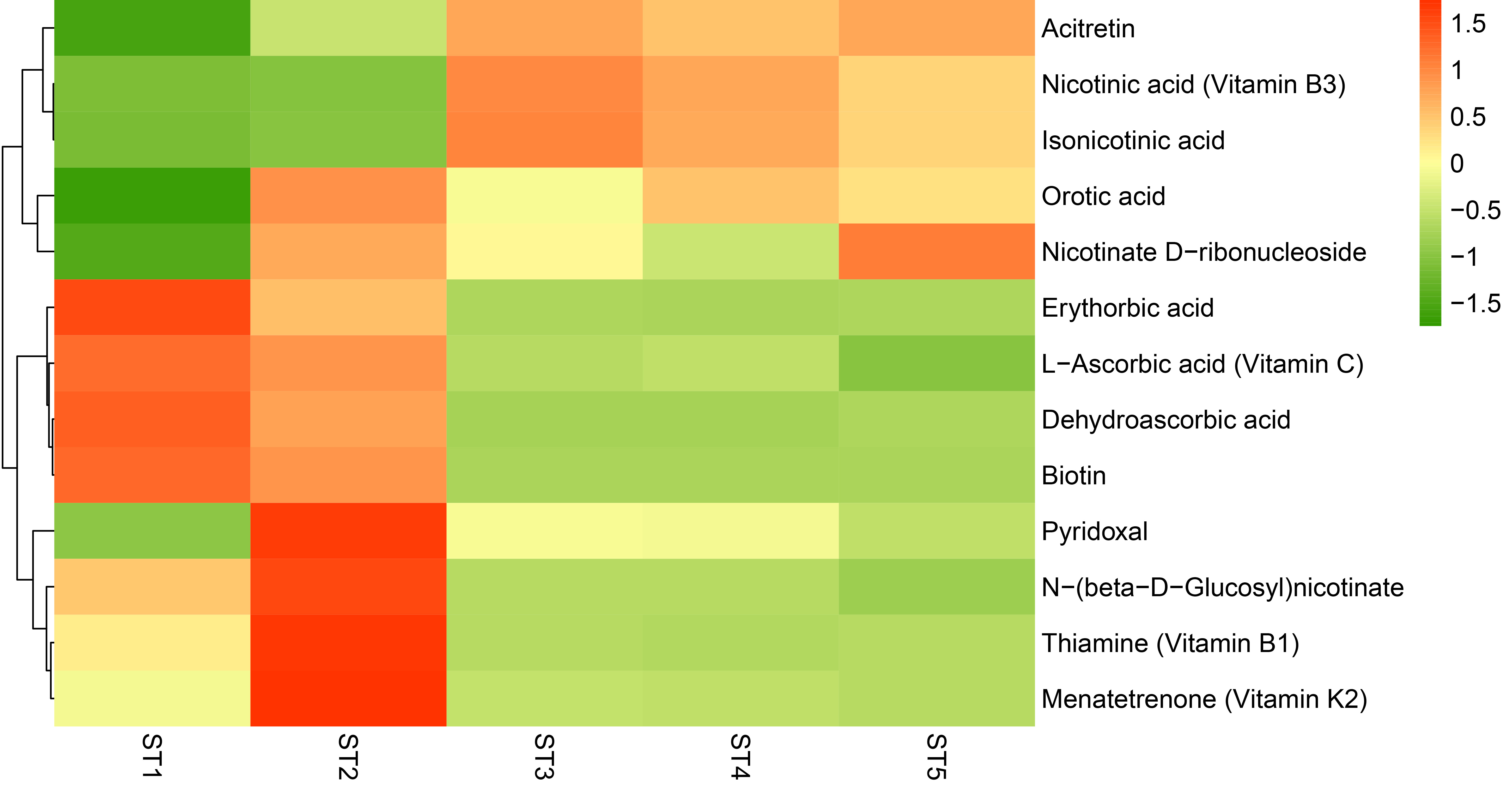

Supplement: Supplementary Figure 1 — Cluster heatmap of 13 differentially accumulated vitamin-related metabolites. [file Image_1.JPEG]

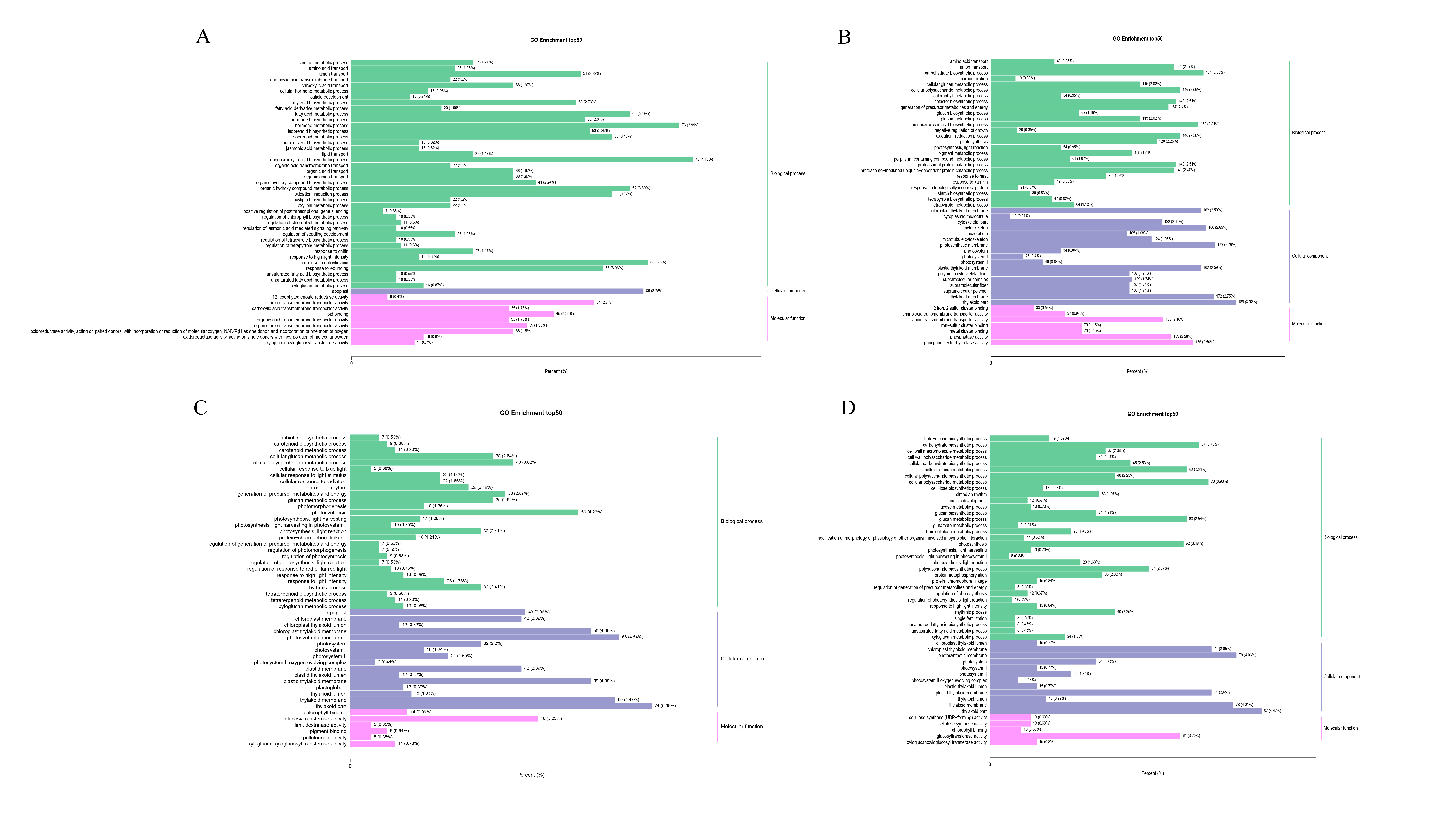

Supplement: Supplementary Figure 2 — Volcano plots of DEGs between different jujube fruit development. The X-axis and the Y-axis represent the fold change of DEGs and the significance level of DEGs, respectively. [file Image_2.JPEG]

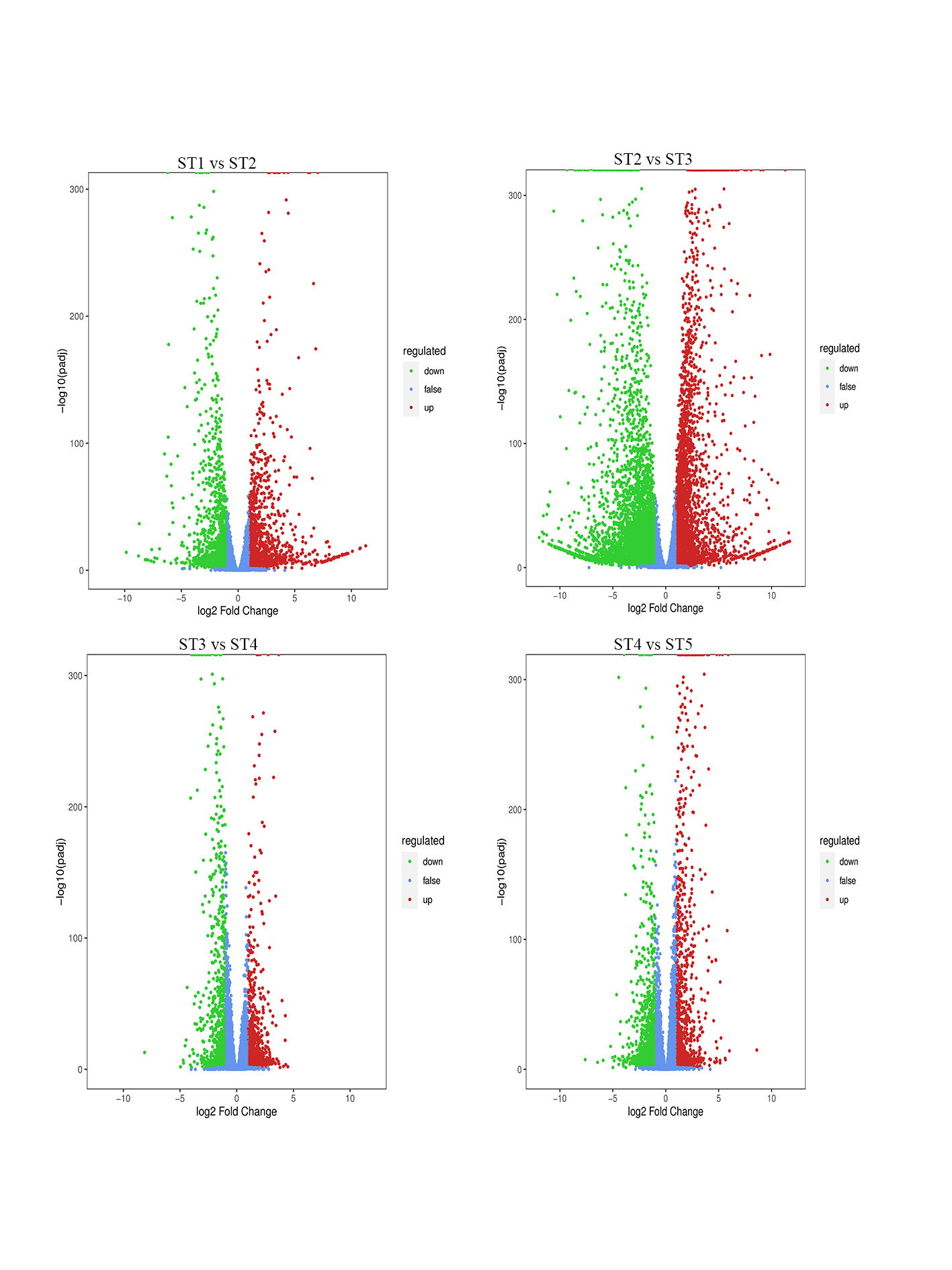

Supplement: Supplementary Figure 3 — GO analysis of DEGs in ST1 vs. ST2 (A), ST2 vs. ST3 (B), ST3 vs. ST4 (C) and ST4 vs. ST5 (D). The X-axis and the Y-axis indicate GO classifications and the number of genes in each classification, respectively. [file Image_3.JPEG]

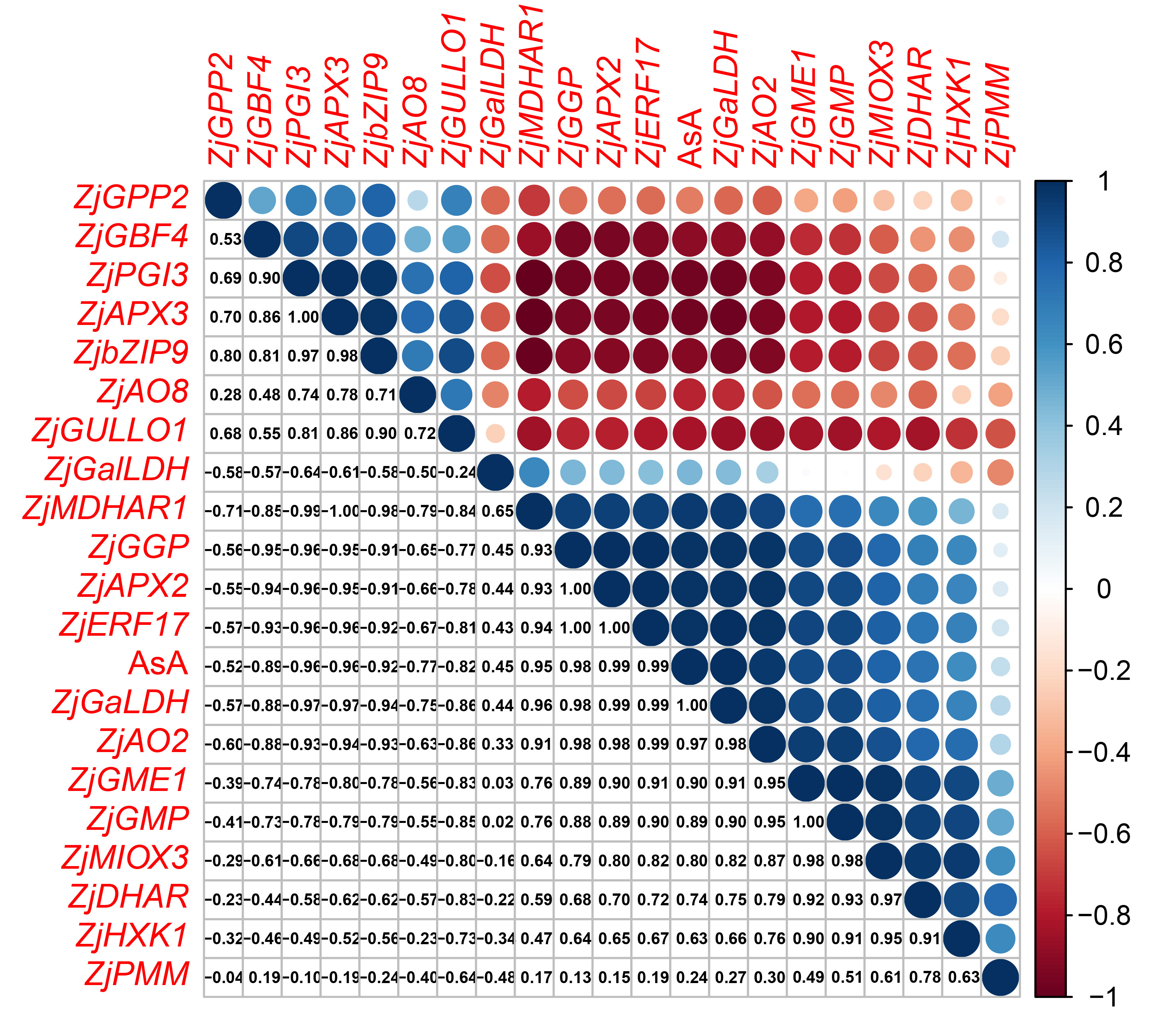

Supplement: Supplementary Figure 4 — Correlation analysis between AsA content and related genes. [file Image_4.JPEG]
